# Dealing with Hidden Threats: The Antimicrobial Effect of the Embalming Process

**DOI:** 10.3390/microorganisms10112180

**Published:** 2022-11-03

**Authors:** Benedict Uy, Simon Swift, Francesca Casu, David Mahuika, Maurice A. Curtis, Deborah Prendergast

**Affiliations:** 1 School of Medical Sciences, Faculty of Medical and Health Sciences, The University of Auckland, 85 Park Road, Grafton, Auckland 1023, New Zealand; 2 Department of Molecular Medicine and Pathology, Faculty of Medical and Health Sciences, Waipapa Taumata Rau, The University of Auckland, 85 Park Road, Grafton, Auckland 1023, New Zealand; 3 Health, Safety and Wellbeing, The University of Auckland, 85 Park Road, Grafton, Auckland 1023, New Zealand; 4 Department of Anatomy and Medical Imaging, Faculty of Medical and Health Sciences, The University of Auckland, 85 Park Road, Grafton, Auckland 1023, New Zealand

**Keywords:** embalming, microbial contamination, antimicrobial, drug-resistant bacteria

## Abstract

Individuals naturally carry bacteria and other microbes as part of their natural flora, with some being opportunistic pathogens. Approximately 30% of the population is known to carry *Staphylococcus aureus* in their nasal cavity, an organism that causes infections ranging from soft tissue abscesses to toxic shock syndrome. This problem is compounded by the presence of antibiotic-resistant strains such as Methicillin-Resistant *Staphylococcus aureus* (MRSA). Commensal bacteria present on cadavers pose a risk to those who handle the body. As a Medical School Anatomy laboratory that performs hands-on cadaveric dissection, we wanted to know whether the embalming process is sufficient to kill all commensal bacteria that pose a risk to staff and students. Even if these strains do not cause disease in these individuals, secondary transmission could occur to friends and family, who may be at higher risk of acquiring an infection. Embalming is assumed to eliminate all microbial contamination on the body. However, there are limited studies to confirm this. This study characterises the incidence of antibiotic sensitive and resistant bacteria in cadavers donated for medical teaching and research. We have screened for Methicillin-Resistant Organisms (MRO) and Extended-Spectrum Beta-Lactamase (ESBL) producing bacteria. In this study group of cadavers, approximately 46% (16/35) carry an MRO, while 51% (18/35) carry an ESBL positive organism prior to embalming. By determining the organisms’ presence pre- and post-embalming, we can evaluate the embalming procedure’s effectiveness. Our results show embalming eliminates detectable microbes in about 51% (18/35) of the cadavers. MRO dropped by 75% (16 to 4 positive cadavers), while ESBL organisms went down by almost 95% (from 18 to 1 positive cadaver). There was a further decrease in the number of positive cadavers after storage at 4 °C to 6% (2/32). Thus, although the embalming process does not immediately sterilise all the cadavers, prolonged storage at 4 °C can further reduce the number of viable bacteria.

## 1. Introduction

The bequest of human bodies enables research and medical teaching. Morticians prepare cadavers by a process of embalming for sanitation and preservation [1]. We assume that the embalming process is fully effective, but if this is not the case more stringent health and safety procedures would be appropriate.

Natural carriage of bacteria is common, particularly as non-pathogenic commensal bacteria. However, many people can asymptomatically carry potentially pathogenic organisms, including drug-resistant strains. Although the bacteria may not be causing disease in the host, many may cause disease when the opportunity arises, such as for patients at greater risk through an existing illness or after surgery. The other concern is the direct transmission of these potential pathogens to more susceptible individuals. Comorbidity and debility can make the treatment required more challenging due to patient factors [2,3]. Infection with antibiotic resistant bacteria can then add another layer of complexity to these issues, particularly due to incorrect or ineffective treatment [4,5]. The transmission of the antibiotic-resistance gene itself to other non-resistant organisms through horizontal gene transfer is also problematic [6]. These transfers are unwanted, as it leads to the spread of antibiotic-resistant bacteria through the community.

Antimicrobial-resistant organisms, or superbugs, are becoming an increasingly common threat. This led former Director General of the WHO, Margaret Chan, to say they “may even bring the end of modern medicine as we know it”. The main issue with these strains are the lack of effective treatment options, leading to the need to use last-resort antibiotics, or less-active antibiotics, that are often associated with worse side effects. The exposure of bacteria to a variety of different antibiotics has led to the development of new resistances and the emergence of multi-resistant, extensively resistant and even totally resistant strains [2,7]. Thus the acquisition of antibiotic-resistant bacteria lead to a worse prognosis for the patient, leading to higher morbidity and mortality rates and extended stays in the hospital [8]. A higher prevalence of these organisms in the community increases the risk of exposure for everyone.

Individuals colonised by antibiotic-resistant bacteria without an active infection are regarded as carriers, and fall into three broad categories: long-term carriers; spontaneous decolonises; and undetermined status [9]. The status can only be confirmed through constant monitoring and is unknown for most people. The long-term carriers present most risk in spreading antibiotic-resistant strains. The major risk-factor for becoming colonised by antibiotic-resistant strains is travel, particularly to an area with a high incidence of antibiotic-resistant bacteria. After initial colonisation, approximately half of the people screened spontaneously decolonise after two months [7]. Six months after travel, approximately 18% of travellers retained antibiotic-resistant strains [10]. Although not detectable, these antibiotic-resistant strains could be present below the limit of detection in these people. Subsequent antibiotic exposure could then provide a selective pressure for these antibiotic resistant strains to expand.

There are many organisms involved in carriage, and their colonisation site varies; for instance, Carbapenem-Resistant Enterobacteriaceae (CRE) are present in the intestinal tract, while penicillin-resistant *Haemophilus influenzae* is found in the upper respiratory tract [11]. Around 30% of the population can carry the opportunistic pathogen *Staphylococcus aureus* in the anterior nares [12]. In a recent surveillance study at a French hospital, almost 40% of the patients who underwent a rectal screening were carrying Vancomycin-Resistant Enterococci (VRE) [9]. These results were similar to a previous hospital study where approximately 26% and 39% of the patients with antibiotic-resistant staphylococcal and enterococcal infections, respectively, had previous colonisation with their respective pathogens [8]. Humans are not the only reservoir of antibiotic-resistant pathogenic microorganisms, as a study of animal husbandry in the United States of America identified various strains of antibiotic-resistant *Salmonella enterica* in many food animals [13]. The spread of antibiotic-resistant organisms through the food chain has not only led to an increase in the acquisition of antibiotic-resistant infections but has also now become a large reservoir for antibiotic-resistant organisms and genes [14].

Many countries have a statutory schedule of notifiable diseases to reduce the transmission of highly pathogenic diseases, but it does not take drug-resistance into account. This lack of notifying incidents of drug-resistance organisms includes the Health Act 1956 in New Zealand or the Public Health (Control of Disease) Act, 1984/the Public Health (Infectious Diseases) Regulations, 1988 in the United Kingdom [15]. In 2017 the WHO, Centre for Disease Control (CDC) and the European Centre of Prevention and Control (ECDC) flagged the serious threat that Carbapenem-Resistant Enterobacteriaceae (CRE), particularly Carbapenemase producing Enterobacteriaceae (CPE), were posing for people exposed. They provided dedicated guidelines for prevention and control of these organisms for health care facilities [16,17,18]. Other countries have joined the effort, for example in 2018 the New Zealand Ministry of Health developed guidelines for infection control and prevention for CPE for hospitals and other health care providers such as rest homes to reduce the burden by CPE infections [19]. The Nelson-Marlborough District Health Board in New Zealand has a guideline on bodies that are safe for embalming based on the risk of infection. The high-risk pathogens are on the schedule of notifiable diseases [20]. However, this is only applicable if the cadaver’s carriage status is known when they arrive for embalming. For the most part, the carriage status of an individual at death is generally unknown. Human Immunodeficiency Virus (HIV), Transmissible Spongiform Encephalopathies, such as Creutzfeldt-Jakob Disease (CJD), and tuberculosis are of the most significant concern to embalmers as the rates of exposure have increased in recent times [15,21]. There are guidelines available to reduce infectious risks directed at healthcare professionals. However, these guidelines are generally aimed towards those who interact with living patients instead of coroners, medical examiners, and morticians who deal with un-embalmed cadavers. These people have the highest risk of colonisation as they are the individuals who come into physical contact with the body and body fluids. The infection risk would also be dependent on the route of transmission as aerosol-based pathogens, such as tuberculosis, would be easier to transmit than those that require blood to blood contact, such as HIV. It would be prudent to have standardised and specific control and preventative measures to prevent the transmission of these organisms throughout the workplace. These controls and preventative measures are generally well established in hospitals but not in other areas, such as in a funeral home. Before embalming, cadavers are generally treated as infectious as a precaution [21].

The embalming of cadavers has two primary purposes, sanitation and preservation [1]. The removal of bacteria colonising the cadaver delays decomposition and putrefaction, allowing the body to retain its structural integrity after death. The risk of infection during the embalming process is high due to the presence of sharp hazards such as needles, scalpels and bones, as well as the continuous exposure to bodily fluids. Embalming fluid is a mixture of chemicals containing formaldehyde or glutaraldehyde, methanol, and other solvents. This mixture fixes cells it comes into contact with, including those of the body’s tissue and the associated microorganisms. The fixation process cross-links proteins or DNA through a reaction with formaldehyde or glutaraldehyde forming a Schiff base. This linkage fixes the tissue into place, keeping the structure and making it harder to break down. Embalming fluid is often commercially available, and the composition may vary depending on the company and the purpose of the embalming. Modern embalming techniques usually involve four methods; arterial embalming, cavity embalming, hypodermic embalming, and surface embalming [22]. These different processes cover the body’s different regions to ensure that it has been completely embalmed. Methods will vary depending on the organisation’s protocols or facility where the embalming is carried out. Generally, embalming a cadaver for long-term preservation for medical research uses the arterial and hypodermic embalming processes as the cavity embalming process damages the chest and abdomen organs by piercing these organs to drain the cadavers’ gas and fluids, and replace it with embalming fluid.

There are limited number of papers looking at pathogens after embalming, as most assume that the body becomes non-infective. Weed et al. (1951) reported a case of viable bacteria, including *M. tuberculosis*, isolated from cadavers after 48 h after embalming [23]. Another study reported the transfer of bacteria from a cadaver onto laboratory coats after a dissection class with a group of medical students [24]. If not adequately treated, then these items of clothing could become reservoirs of bacteria. If these cadavers were colonised with viable antibiotic-resistant bacteria, then a chain of infection may occur if transmitted between student, staff, and other members of the public. Cases of direct pathogen transmission are the worst-case scenario for embalmers, and Sterling et al. detail an embalmer acquiring an active *M. tuberculosis* infection after embalming a cadaver of a person known to be infected with *M. tuberculosis* [25]. The assumption of bacteria free cadavers after embalming may not be correct, and so the confirmation of the bacterial load of cadavers post-embalming is an important health and safety issue for those that encounter an embalmed cadaver, such as morticians and medical students. This study aims to determine how well the embalming process eliminates commensal and antibiotic-resistant micro-organisms from cadavers.

## 2. Materials and Methods

### 2.1. Cadavers

Human body donation is administered by the Human Body Bequest Programme, through the Department of Anatomy and Medical Imaging, at the University of Auckland, New Zealand, for medical research. The process of accepting and managing bequests is governed by the Human Tissue Act 2008 and is carried out under the formal authority and guidance of the Inspector of Anatomy, New Zealand Police. Body donation can only occur with the consent of the family. Obtaining donors’ medical history and infectious disease status is an integral part of determining the body is appropriate for donation. Upon arrival, the body is embalmed using the Common Carotid artery within 24 h for long-term preservation. The cadaver was then sealed in two sperate plastic cadaver bags and stored at 4 °C until required for teaching. Cadavers embalmed use approximately 20 L of formaldehyde based Anatomical Embalming Fluid (Regal Manufacturers Limited, Wellington, New Zealand) through arterial embalming. The actual volume used for embalming is dependent on the body type/condition.

### 2.2. Sample Collection

With the University of Auckland, Biological Safety approval (B02146), pre-embalming samples of the cadavers were collected on arrival at the Human Anatomy Laboratory, The University of Auckland. Sample processing was performed in the University of Auckland PC2 laboratories. Post-embalming samples were also collected 24 h after the embalming process and prior to use for medical teaching. It should be noted that subsets of cadavers were swabbed at each sampling period as their required research usage varied, but a core set (n = 29) was swabbed at all three-time points. Sterile swabs were used to acquire samples from the cadavers pre- and post-embalming. Each cadaver’s nasal vestibule and anal canal were swabbed. After swabbing, the swabs were placed into 10 mL of sterile peptone water (Fort Richard) before storage at 4 °C until required, varying from days to weeks post isolation. Blood (5–12 mL) was collected by cardiac puncture. After collection, the blood was stored at 4 °C until required. Blood samples were only available for pre-embalming as the embalming fluid replacing the blood during the embalming process.

### 2.3. Sample Plating

Once collected, samples were spread-plated onto a series of selective and differential agars listed in Table 1. The CHROMagars used are selective for specific organisms and are able to differentiate between the organisms grown based on the colour of the colonies that grow (Table 2). The horse blood agar was not selective and should support the growth of a broad range of microorganisms. The Sabouraud dextrose agar is a selective agar for fungi but is unable to differentiate between different fungal species. Agar plates were prepared as directed from powder, apart from the pre-prepared horse blood agar (Fort Richard, Auckland, New Zealand). Briefly, samples were diluted 1:10 with sterile PBS. 100 µL of the diluted and neat sample was spread plated onto appropriate agar plates and incubated at 37 °C overnight. The dilution was to acquire single colonies for identification, but not for enumeration. Microbial growth was recorded, and plates were stored at 4 °C until they were photographed. For agar plates without antibiotics a positive growth result was recorded if there were five or more colonies present. For agar plates with antibiotics (ESBL and MRSA) a positive growth result was recorded for the presence of one or more colonies.

### 2.4. Sample Collection over Time

The same sampling procedure was performed on the cadavers once they were assigned for teaching. These cadavers were stored at 4 °C between embalming and the start of the teaching year. The post-embalming time between the different cadavers varied from two months to 14 months. The purpose of this second set of testing was to determine the effects of embalming over time. These samples were plated onto non-selective horse blood agar, with a positive growth result recorded for the presence of 5 or more colonies.

## 3. Results

### 3.1. Cadaver Information

**Figure 1:**
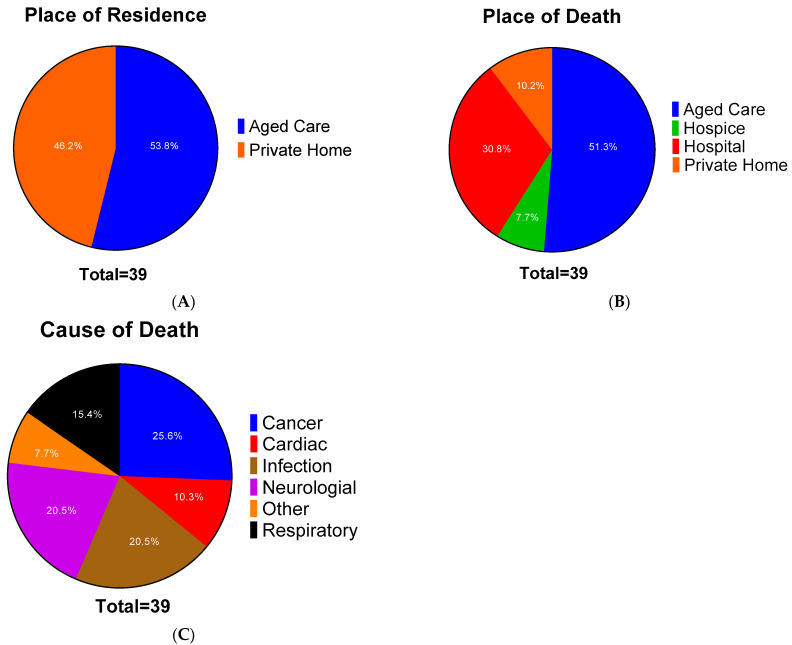
Pie charts showing the distribution of residence (**A**), place (**B**), and cause of death (**C**). Pie charts that show the distribution of the different metrics. The number in the segments shows the percentage. n = 39.

The cadavers were collected throughout the year for the study. In total, data was collected from 39 cadavers. Not all cadavers were used in all the different screening processes from this pool. The cadavers’ average age was 83, while the median was 86 with a minimum age of 56 and a maximum age of 99. The gender split was 51.3% (20/39) male and 48.7% (19/39) female. Figure 1 gives the place of residence (Figure 1A), the place of death (Figure 1B) as well as the cause of death (Figure 1C). This information was acquired from the HP4720 Medical Certificate of Cause of Death.

The place of residence was split evenly between private homes (46.2%–18/39) and in aged care facilities (53.8%–21/39). Regarding the place of death, over half of these cadavers (51.3%–20/39) were from aged care homes. The next largest group was from the public hospitals, at 30.8% (12/39). The remaining data was split between at private homes (10.2%–4/39) or in a hospice (7.7%–3/39). Likely, those staying in the aged care facilities died there, while the majority of those who resided in private homes were moved prior to death. This move prior to death is not surprising, as many aged care facilities have hospitals on site.

The most typical cause of death was cancer-related at 25.6% (10/39) of the cadavers. The following common cause of death was infectious disease and neurological disorders, both at 20.5% (8/39). Pneumonia and other infectious causes such as endocarditis were grouped as infectious diseases rather than the organ system involved. It is important to bear in mind that many of these people had comorbidities that would have contributed to their deaths due to their age. In our sample, 84.6% (33/39) had known comorbidities, while 2.6% (1/39) had none, and 12.8% (5/39) were not stated.

### 3.2. Bacterial Screening

**Figure 2:**
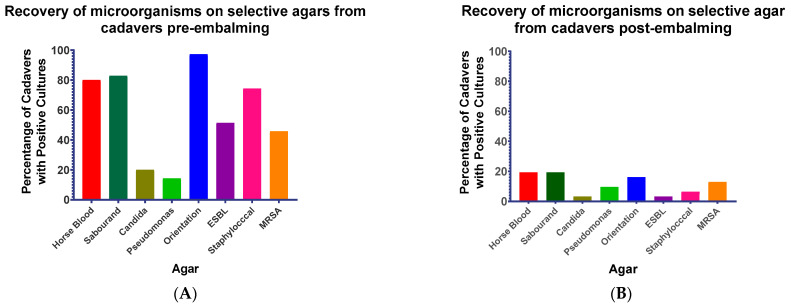
Growth of different samples on different agar plates. The numbers show the number of plates with growth for each patient, regardless of sample type. n = 35 for pre-embalming (**A**) and n = 31 for post-embalming (**B**). Post-embalming samples were taken 24 h after the embalming process.

A nasal swab, anal swab and blood sample was taken from each cadaver pre-embalming. A nasal swab and anal swab were taken from each cadaver post-embalming. Each sample was plated onto a series of different plates to determine what organisms were present. The number of cadavers returning positive results for any of the samples is shown in Figure 2. Each of the agars used returned a positive result for at least one cadaver and for samples taken pre-and post-embalming.

Pre-embalming 34/35 cadavers returned at least one positive sample. Anal swabs returned positive samples from almost all cadavers (Table 3), with nasal swabs and blood samples also returning numerous positive samples (Table 3).

Orientation plates representing Gram-negative bacteria, predominantly from the gut, returned positive results from 34/35 cadavers. A high number of positive samples was returned for SDA (29/35); HBA (28/35) and Staphylococcal (26/35) plates, with much lower rates returned for Candida (7/35) and Pseudomonas (5/35) plates (Figure 2A).

Embalming gave a substantial reduction in the number of cadavers returning positive nasal swab or anal swab samples (18/31), but micro-organisms were not eliminated (Figure 2B). The cadavers returned 5/31 orientation plate, 1/31 ESBL, 6/31 SDA, 6/31 HBA, 2/31 Staphylococcal, 4/31 MRSA, 1/31 Candida and 3/31 Pseudomonas positive samples.

### 3.3. Determination of Antibiotic-Resistant Bacteria on the Cadavers

A focus of this investigation was the evaluation of antibiotic-resistant strains, ESBL and MRSA, present on cadavers pre- and post-embalming. We used agar plates able to select and differentiate species producing ESBLs and select and differentiate MRSA strains. We compared the cadavers positive for ESBL with those positive for growth on the orientation plates that grew Gram-negative gut bacteria. We compared the cadavers positive for MRSA with those positive for growth on the Staphylococcal agar. ESBL and MRSA plates returned lower positive samples from the pre-embalming cadavers compared to their non-resistant counterparts (Table 4).

Of the 35 cadavers screened pre-embalming, 51.4% (18/35) screened positive for ESBL, while 45.7% (16/35) of the cadavers showed the presence of a methicillin resistant organism (MRO) by growth on the MRSA plate. The results imply a high colonisation rate of antibiotic-resistant strains in our sample group. Only one of the cadavers had ESBL growth from all three samples, while six had growth from two of the three samples. Three of the cadavers with growth on the MRSA plates showed growth from two samples. The cadavers showing growth from the anal and/or nasal swabs were presumed to be colonised by the antibiotic-resistant bacteria before death.

**Figure 3:**
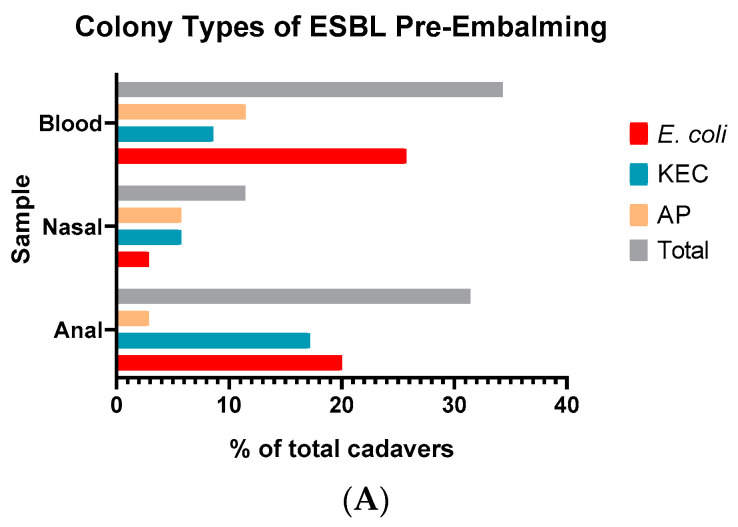

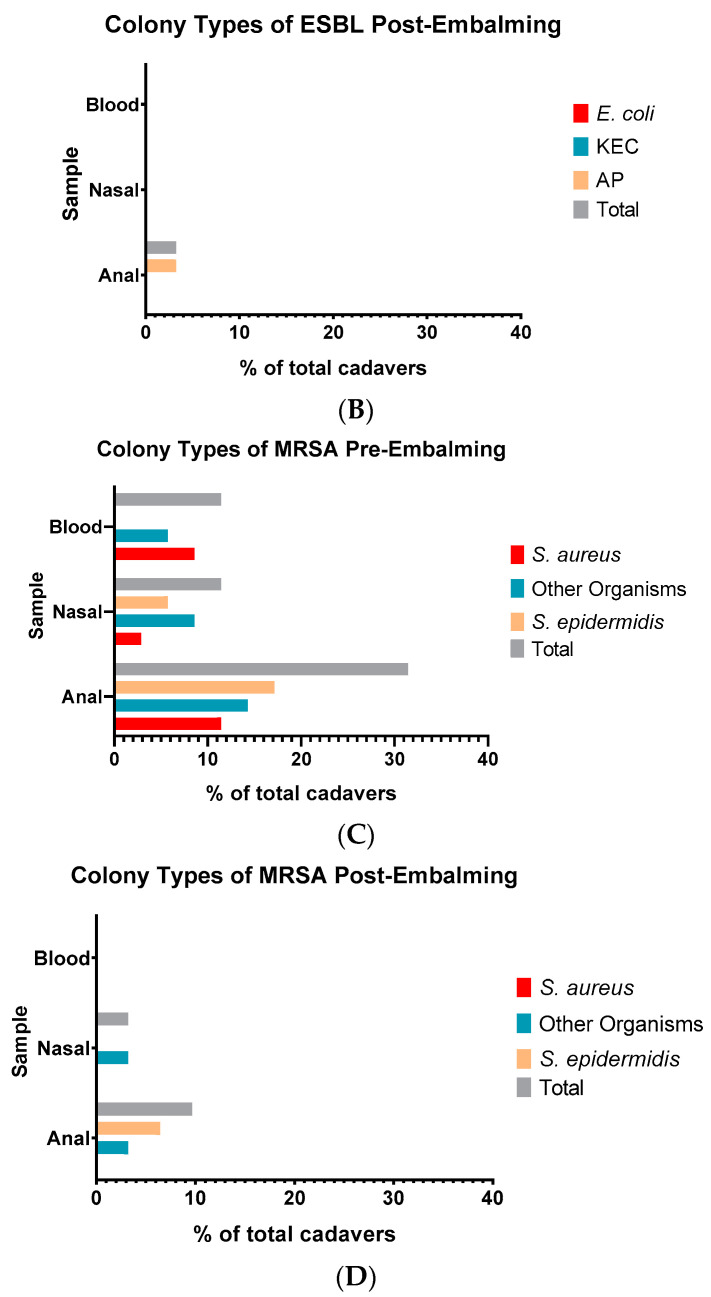
Differentiation of antibiotic-resistant strains. Percentages were determined based on n = 35 pre-embalming (**A**,**C**) and n = 31 for post-embalming (**B**,**D**). The total number is the number of cadavers with growth on the plates. Some sites had growth with different coloured colonies on a single plate. For ESBL plates (**A**,**B**), *E. coli* are the pink/red/purple colonies, KEC (Klebsiella, Enterobacter, and Citrobacter) are the green/purple colonies. AP (Acinetobacter and Pseudomonas) are the cream/translucent colonies. For MRSA plates (**C**,**D**), *S. aureus* is the pink/red/purple colonies (MRSA), the other organisms are the green/blue colonies, and *S. epidermidis* is the cream/translucent colonies (MRO).

We were able to isolate an ESBL organism from 34.3% (12/35) of the blood samples. Of these, 29.7% (9/35), were the pink/red/purple colonies indicating the isolation of *E. coli* (Figure 3A). There was a similar level of green/blue and cream/colourless colonies which differentiate for *Klebsiella*, *Enterobacter* species and *Acinetobacter*/*S. epidermidis* respectively. The levels were slightly lower for MRO (Figure 3C). Interestingly, we did not isolate any cream/colourless colonies on the MRSA plates which would have identified Methicillin resistant *S. epidermidis*.

Nasal samples had an even spread between the different ESBL and MRO species. Based on the location, from the ESBL plates, the green/blue organisms possibly indicated *Klebsiella* species, while the cream/colourless colonies were *Acinetobacter* species.

We isolated 31.4% (11/35) of EBSL organisms in the anal samples. The anal samples were where we isolated the highest number of MRO at 31.4% (11/35). Most of the organisms which were ESBL positive were either pink/red/purple or green/blue, isolated at 20% (7/35) and 17.1% (6/35) respectively. On the MRSA plates, most of the organisms that were isolated in anal samples were cream/colourless which indicates isolation of *S. epidermidis*.

We see a marked reduction in isolatable organisms post embalming (Figure 3B,D). We were only able to isolate an ESBL organism from 3.2% (1/31) of the samples. For MRSA, this was higher at 12.9% (4/31). Although it has reduced from the pre-embalming figures, it indicates that some of these antibiotic-resistant strains could still be present after the embalming process.

### 3.4. Effect of Time on Cadaver

**Figure 4:**
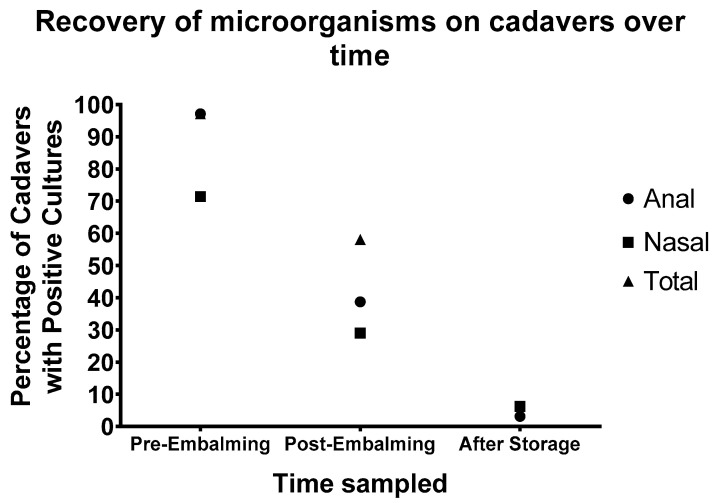
Effect of microbial growth on embalmed cadavers over time. Cadavers were swabbed multiple times in the same areas, following different points in the embalming process. Pre-embalming is when the cadavers arrive at the facility (n = 35). Post-embalming sample collection occurred 24 h after embalming was performed (n = 31). After storage, the cadavers were removed from 4 °C and stored in the teaching laboratory (n = 32). A positive cadaver is one that has had growth in any of the agar plates for the sample. The total is the number of cadavers with growth from either anal or nasal samples.

At the University of Auckland Human Anatomy Laboratory, the cadavers are collected over the year for use the following year. After embalming, they are stored at 4 °C until they are required. To investigate the long-term effect of embalming on microbial carriage, the cadavers were re-swabbed on being removed from cold storage for the new teaching year. Thirty-two cadavers were rescreened, and anal swab, nasal swab and total swab positive cadavers were counted (Figure 4). It is clear that just as embalming reduced the number of cadavers from which viable bacteria could be recovered after 24 h, prolonged storage at 4 °C further reduced the number of cadavers testing positive (Figure 4). Nevertheless, 6.25% (2/32) returned positive samples.

Only anal and nasal are investigated, as blood samples are not available post-embalming. Pre-embalming, there was 97.1% (34/35) of the cadavers with growth in anal samples and 71.4% (25/35) growth in nasal samples. The pre-embalming total is the same as the number of anal samples, 97.1% (34/35) since all cadavers with detectable growth were for anal samples. The percentage of positive cadavers reduced to 38.7% (12/31) and 29% (9/31) for anal and nasal samples, respectively post-embalming. These totals show the number of cadavers with any detectable growth. Although the number of positive cadavers post-embalming seem high, we observed that the positive plates were generally from one site or the other, and not both as was seen in pre-embalming cadavers. Only 9.68% (3/31) of the cadavers had growth both in anal and nasal samples.

Long-term microbial growth was reduced further to 3.1% (1/32) and 6.3% (2/32), for anal and nasal samples, respectively, after being left in storage before usage. This implies that the formaldehyde in the cadavers were continuing to have an effect, even after the embalming process. Of the cadavers with detectable growth after prolonged storage, one of the cadavers was only screened after storage and did not have any pre-or post-embalming data. The other cadaver had no growth post-embalming but did have some after storage. This result indicates that prolonged storage could also lead to recontamination of the cadavers, but more research would be required to determine if this is the case.

### 3.5. Carriage of Antibiotic Resistant Bacteria Prior to Death

**Figure 5:**
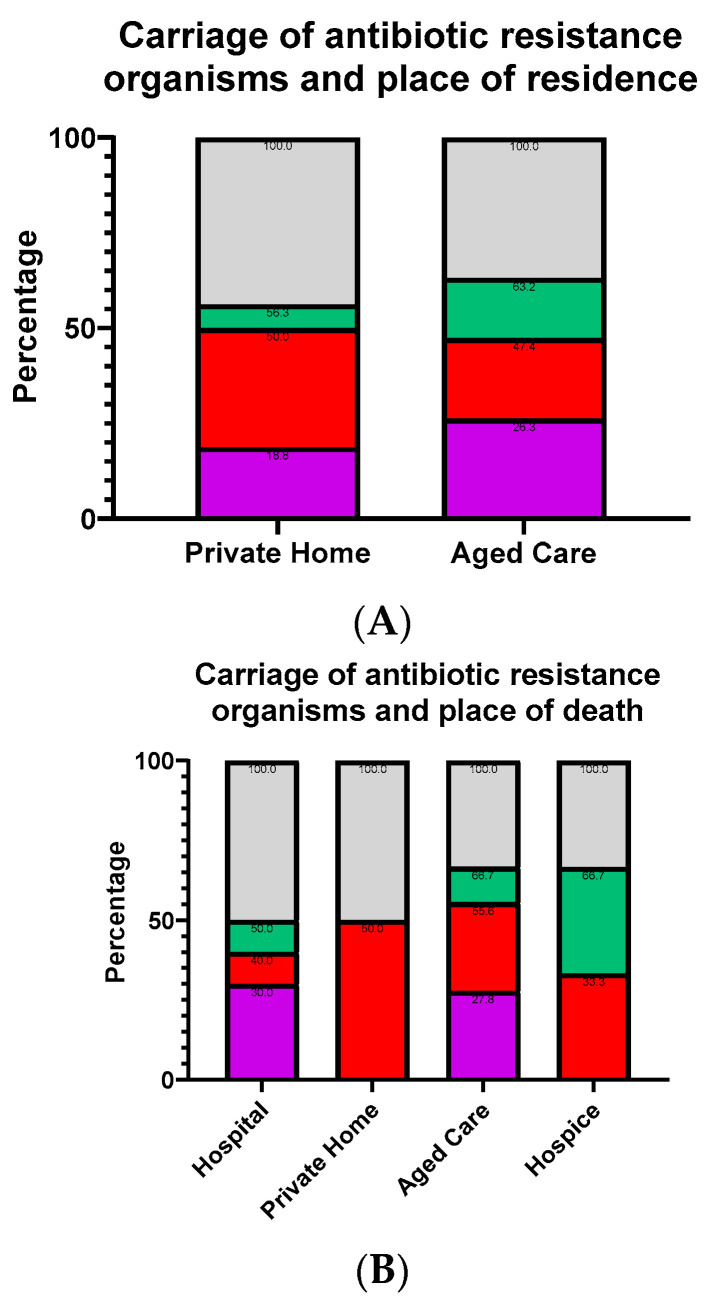

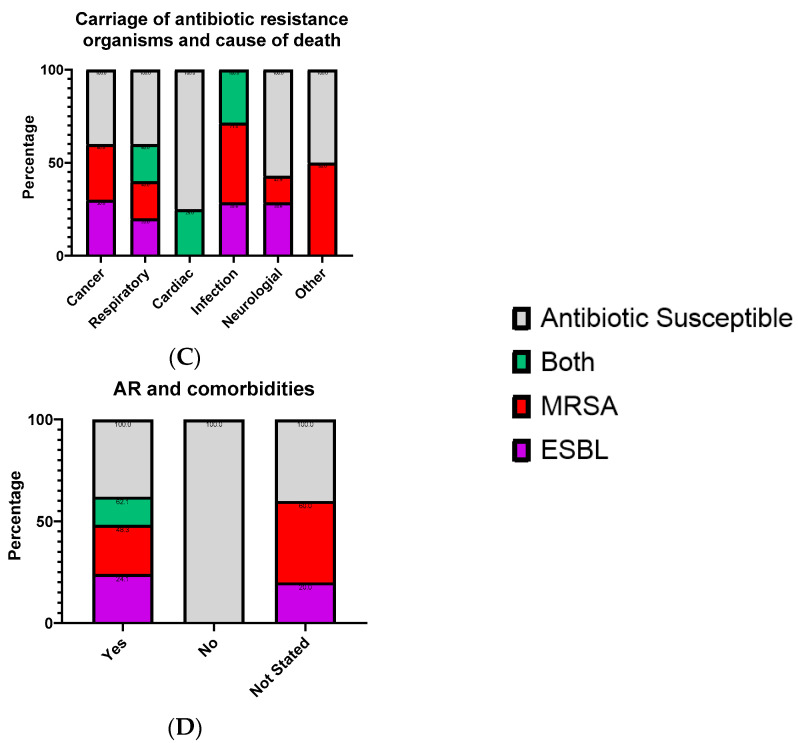
Antibiotic resistance carriage in respect to other factors. The figures show the distribution of antibiotic resistant organisms based on their place of residence (**A**), place of death (**B**), cause of death (**C**) and comorbidities (**D**). This data utilised the set of cadavers (n = 35) with growth of antibiotic resistant bacteria on the pre-embalming plates. The numbers within the segments show the additive proportion from each segment and those below it.

Following the identification of the cadavers with antibiotic resistant bacteria, we looked at the relationship between antibiotic resistance and the cadaver information gathered in Figure 1. The percentage of antibiotic resistant bacteria of the cadavers were compared between the different sets of information (Figure 5). We initially looked at the place of residence (Figure 5A), comparing between private homes and aged care and the distribution of antibiotic susceptible, MRSA and ESBL. Both MRSA and ESBL were similar between the two groups. We isolated antibiotic resistant organisms in over half of the cadavers in this study. This spread was fairly similar regardless of place of residence, but the aged care residents had a slightly higher incidence at 63.2% of the cadavers carrying an antibiotic resistant organism, compared to the 56.3% of those from private homes (Figure 5A). There was a higher number of people in aged cared carrying ESBL, 26.3% vs. 18.8%, and more than twice as many people who carried both MRSA and ESBL at 15.8% compared to 6.25%. When looking at the place of death (Figure 5B), only cadavers from hospital and aged care had detectable ESBL.

When looking at the cause of death (Figure 5C), all cadavers of those whose disease or condition leading to death was due to infections, returned positive samples for either/or both of ESBL and MRSA. This does not indicate the organisms were the cause of death but may represent either colonisation in the place of treatment, or the result of selective pressure from antibiotics treating the illness. The remaining cause of deaths was evenly spread between the different antimicrobial resistant organisms as well as the antibiotic susceptible organisms. The comorbidities (Figure 5D) also show a fairly even spread of the different organisms in the presence of comorbidities. It is important to note that even though there seems to be a high proportion of antibiotic resistant organisms in those with non-stated comorbidities, as stated before, due to their age they most likely had comorbidities, however, were not documented. There were also very few people, 14.3% (5/35), with unknown comorbidities which may have skewed the results.

## 4. Discussion

The common belief is that the embalming process kills all the bacteria by chemically sterilising the cadaver. Only a few studies have investigated bacterial viability after embalming. Weed et al. (1951) was able to isolate multiple pathogenic organisms from different tissues 24–48 h post-embalming, including *Mycobacterium tuberculosis* [23]. A more recent paper has reported the isolation of a series of bacteria from multiple anatomical regions post-embalming [26]. Balta et al. (2018) looked at the antimicrobial effects of different embalming fluids [27]. The method of embalming performed was not specified in most of these papers. Different embalmers would presumably perform the process with different protocols, affecting the embalming fluid distribution and the overall efficacy of the embalming process. The cadavers in this study were embalmed for long-term preservation using arterial and hypodermic injection using the Common Carotid artery, while blood was drain from the corresponding Jugular vein. All cadavers were embalmed by the same embalmer. Embalming a cadaver for long-term preservation uses more embalming fluid and formaldehyde to sanitise the body. It may be that some unrecognised variability in the cadavers can explain why some remained positive for the presence of microbes. It is also possible that the superficial sites sampled (nasal passage and anal passage) are exposed to lower levels of embalming agents and some bacteria may survive. Knowing the bacterial load prior to embalming gives us an idea of the risk of exposure for the people who encounter the cadavers, such as the general medical staff attending the deceased and the funeral homes delivering the donated bodies to the medical school. The growth present on the cadavers post-embalming gives us an approximate idea of the microbial load present on these cadavers so that we can assess the risk for those who then come into contact with them such as medical students and morticians.

Another important aspect is the embalming fluid used. The ideal embalming fluid would preserve the body and keep the same features as if they were not embalmed [28]. Embalming fluids are a cocktail of chemicals that aims to sanitise and preserve a decomposing body [1]. There are many different solutions available depending on usage and may work at different rates due to chemical composition. Formaldehyde alone can make the cadaver inflexible, limiting its uses. Other methods have been developed to make the embalmed cadaver look more natural such as Thiel embalming for preserving colour [29]. The index (percentage of preserving chemicals) is dependent on the purpose. For instance, cadavers embalmed for repatriation to their home countries would use a higher index solution than one for a funeral home. Embalming for teaching also uses a high index, similar to embalming for repatriation. The embalming fluid used for long-term preservation for teaching has a formaldehyde concentration between 50–70%. In light of our findings, it may be that lower index embalming fluids that focus more on preserving the natural structures for presentation to the family may leave an unwanted microbial burden on the cadaver, but further tests will be required to determine if this is the case.

In this study we determined that even if people carry antibiotic-resistant bacteria as part of their normal flora, these bacteria’s levels are generally lower than the total number of isolatable bacteria. This can be seen in the difference between the number of positive Orientation/ESBL plates and the Staphylococcal/MRSA plates as the only difference between the plates is the presence of antibiotics. Interestingly, 75% (12/16) of the growth from blood samples was ESBL positive, indicating that these antibiotic-resistant strains may play a substantial role in septicaemia [30,31,32]. This higher incident of ESBL positive bacteria in the blood samples may be due to the wide diversity of ESBL -producing pathogens in our community [33] and the reported prevalence of *K. pneumoniae* isolates [31]. However, the prevalence of ESBL in New Zealand over the past decade is at <5% and the prevalence of ESBL *K. pneumoniae* bloodstream isolates is between 10–15% [34]. Our high incident of ESBL growth from blood samples would be expected due to the demographics of our cohort.

Although the focus is primarily on cadavers, this study has shown that over half of the elderly population (53.8%) reside in aged care facilities and of these, 63.2% were carrying antibiotic resistant bacteria. This implies that those working in aged care facilities could potentially have higher rates of exposure towards antibiotic resistant organisms, particularly the caregivers who spend the most time with the residents [35]. Most infections are spread by direct contact from an infected person’s bodily fluids or indirectly by contact with contaminated equipment or surfaces such as hospital sinks and taps [36,37,38].

Balta et al. were not able to isolate bacteria post embalming, even directly after the embalming process [27]. However, the sample size in that paper was very small with two cadavers for each treatment. In our larger sample size of 35 cadavers, 18 of the 35 (51.4%) also showed no growth directly after embalming. We were able to pick up the presence of bacterial growth post-embalming due to our large sample size. The treatment of the cadavers pre-embalming may also have had an effect as this study embalmed fresh cadavers whereas Balta et al. froze their cadavers at −20 °C prior to embalming, which may have caused a hurdle effect on the commensal microbial population prior to embalming. Although we were still able to isolate antibiotic-resistant bacteria after embalming, the levels were relatively low at 3.2% (1/31) for ESBL strain and 12.9% (4/31) for MRO. Although these organisms were not MRSA, they are still methicillin-resistant and important, as they could pass their resistance genes to other organisms [39]. The differential CHROMagar plates’ properties can give us an idea of which organism might be involved; it does not verify the species. Using these plates supplemented with other methods would have helped identify these antibiotic-resistant organisms; e.g., 16S rRNA sequencing [40]; mass spectroscopy [41], specific PCR amplicons [42], and commercial kits to test phenotypic and biochemical properties [40,43]. Although an extra step, the identification process could add valuable data, particularly in identifying specific organisms.

After the initial reduction of microbial numbers post-embalming, there was a further reduction after the cadavers were stored at 4 °C. The same person sampled the cadaver’s limiting variability at this step. For the embalming fluid to be effective, it needs to penetrate through tissues to fix extravascular areas. Another critical factor is the speed of fixation. The different embalming agents vary in these aspects. For instance, formaldehyde is quite penetrative but slow-acting [1]. Cold storage of the cadavers before use may have given the embalming fluids extra time to penetrate further and fix the cadaver, producing a prolonged embalming effect on the body, which may account for the microbial reduction observed. Alternatively, the storage process may have provided an extra stress on the bacteria that had initially survived the embalming process leading to their elimination. Further investigation is required to determine the cause of the microbial reduction after cold storage post-embalming.

An interesting finding with the long-term storage is that the cadavers themselves could become contaminated with microorganisms. One of the two samples with growth after long-term storage did not have detectable growth post-embalming. This implies that a contamination event may have occurred on the cadaver between storage after embalming and removal after storage. Although bacteria can be recovered from embalmed cadavers, many were determined to be commensal bacteria [26]. These bacteria could have survived the embalming process or passed on by living personnel after being embalmed. The issue would come if antibiotic-resistant bacteria or other pathogenic organisms are passed onto and contaminates the cadavers. The cadaver can then become a reservoir of these unwanted organisms. The sample size with growth after the storage is low (n = 2), so further testing would need to be performed to confirm embalmed cadavers’ contamination.

One of the most significant limitations of this project is the restriction on enumeration of the microbes that have been isolated. Although there appeared to be a wide diversity of microbial strains from all three sampling sites. This limitation meant we were unable to quantify the level of microbial reduction due to the embalming process. What was prominent was that many of the plates pre-embalming had confluent growth, while the majority of the plates with growth seen post-embalming were down to countable colonies (less than 500 colonies). The embalming process reduced the number of positive sites of the cadavers, as growth was mainly detected either in anal or nasal samples instead of both, which further suggests a reduction in the microbial load after embalming. As we are only looking at culturable bacteria, we are not taking into account those which are unculturable such as the obligate anaerobes. A metagenomic method such as transposon aided capture (TRACA) could also be a method to determine the presence of antibiotic resistance genes when coupled with sequencing [44].

## 5. Conclusions

The embalming process reduces the microbial contamination to below the detection limit in about half of the cadavers embalmed. Reduced microbial growth extended to over 90% of the cadavers in the study after storing the embalmed cadaver at 4 °C for at least another month. Although it is not as instantaneous as expected, the embalming procedure reduced the cadavers’ microbial counts. A small proportion of the antibiotic-resistant bacteria survived the initial embalming process but were no longer detectable after storage. The fact that the embalming and storage process reduced the detectable microbial load on cadavers can be used to inform risk assessments in medical teaching and research settings to improve health and safety of these workplaces.

## Figures and Tables

**Table 1:** Microbiological agar used for the study.

Agar	Source	Organism Selected	Sample
Candida Chromagar	BD Difco	Candida	Nasal
ESBL Chromagar	Chromagar	ESBL positive Gram-Negative Organisms	Anal, Nasal, and Blood
Horse Blood	Fort Richard	Non-Specific	Nasal and Blood
MRSA Chromagar	Chromagar	Methicillin Resistant halophile	Anal, Nasal, and Blood
Orientation Chromagar	Chromagar	Gram-Negative organisms	Anal, Nasal, and Blood
*Pseudomonas* Chromagar	Chromagar	Pseudomonas	Nasal
Sabouraud Dextrose	BD Difco	Fungi	Anal and Nasal
Staphylococcal Chromagar	Chromagar	Halophile	Anal, Nasal, and Blood

Note: The orientation and Staphylococcal Chromagar are the same as the ESBL and MRSA Chromagar, respectively, and without their respective antibiotics.

**Table 2:** Colour of Chromagar for differentiation.

Media	Colour	Organism
Candida Chromagar	Grey/Green	*Candida albicans*
	Mauve	*Candida glabrata*
	Pink and fuzzy	*Candida krusei*
	Blue	*Candida tropicalis*
ESBL/Orientation Chromagar	Cream	*Acinetobacter*
	Pink/Red/Purple	*Escherichia coli*
	Blue/Green	*Klebsiella*, *Enterobacter*, *Citrobacter* and *Serratia*
	Translucent (Cream to green)	*Pseudomonas*
	Turquoise Blue	*Enterococcus*
	Cream, pinpoint	*Staphylococcus epidermidis*, *Candida albicans*
MRSA/Staphylococcal Chromagar	Pink to Mauve	*Staphylococcus aureus*
	White/Translucent	*Staphylococcus epidermidis*
	Blue/Green	Other bacteria (not specified)
Pseudomonas Chromagar	Blue to Green	*Pseudomonas*
	Violet/Translucent	*Klebsiella pneumoniae*

The media used enabled an approximation of the bacteria isolated. Due to the restrictions imposed by the Biological Safety Committee, further investigations regarding identify the isolated organisms was not possible.

**Table 3:** Distribution of bacteria from the different sample sites.

Agar	Anal Samples	Nasal Samples	Blood Samples
Candida Chromagar	14.3%	14.3%	-
ESBL Chromagar	31.4%	11.4%	34.3%
Horse Blood	-	68.6%	57.1%
MRSA Chromagar	31.4%	11.4%	11.4%
Orientation Chromagar	97.1%	65.7%	45.7%
*Pseudomonas* Chromagar	-	14.3%	-
Sabouraud Dextrose	80.0%	40.0%	-
Staphylococcal Chromagar	57.1%	42.9%	17.1%

The number shows the percentage of cadavers with growth on the plate from a specific site pre-embalming. n = 35. “-” denotes no samples taken for that plate.

**Table 4:** Counts of antibiotic-resistant bacteria from the different sample sites.

Agar Plate	Orientation	ESBL	Staphylococcal	MRSA
Growth from Anal Samples	34	11	20	11
Growth from Nasal Samples	23	4	15	4
Growth from Blood Samples	16	12	6	4

The number shows the number of cadavers with growth on the plate from a specific site pre-embalming. n = 35. Note that it is likely that the bacteria, which have grown on the antibiotic plate (ESBL and MRSA), would most likely also have grown on their non-antibiotic counterpart (Orientation and Staphylococcal, respectively).

## Data Availability

Not applicable.

## References

[1] Ajileye A.B., Esan E.O., Adeyemi O.A. (2018). Human Embalming Techniques: A Review. Am. J. Biomed. Sci..

[2] Fair R.J., Tor Y. (2014). Antibiotics and bacterial resistance in the 21st century. Perspect Med. Chem..

[3] Videcnik Zorman J., Lusa L., Strle F., Maraspin V. (2013). Bacterial infection in elderly nursing home and community-based patients: A prospective cohort study. Infection.

[4] Peralta G., Sánchez M.B., Garrido J.C., De Benito I., Cano M.E., Martínez-Martínez L., Pía Roiz M. (2007). Impact of antibiotic resistance and of adequate empirical antibiotic treatment in the prognosis of patients with *Escherichia coli* bacteraemia. J. Antimicrob. Chemother..

[5] Zarkotou O., Pournaras S., Tselioti P., Dragoumanos V., Pitiriga V., Ranellou K., Prekates A., Themeli-Digalaki K., Tsakris A. (2011). Predictors of Mortality in Patients with Bloodstream Infections Caused by KPC-Producing *Klebsiella pneumoniae* and Impact of Appropriate Antimicrobial Treatment. Clin. Microbiol. Infect..

[6] Levin B.R., Lipsitch M., Perrot V., Schrag S., Antia R., Simonsen L., Walker M., Stewart F.M. (1997). The population genetics of antibiotic resistance. Clin. Infect. Dis..

[7] Basak S., Singh P., Rajurkar M. (2016). Multidrug Resistant and Extensively Drug Resistant Bacteria: A Study. J. Pathog..

[8] Gleason T.G., Crabtree T.D., Pelletier S.J., Raymond D.P., Karchmer T.B., Pruett T.L., Sawyer R.G. (1999). Prediction of poorer prognosis by infection with antibiotic-resistant gram-positive cocci than by infection with antibiotic-sensitive strains. Arch. Surg..

[9] Farfour E., Si Larbi A.G., Couturier J., Lecuru M., Decousser J.W., Renvoise A., Faibis F., Lawrence C., Nerome S., Lecointe D. (2020). Asymptomatic carriage of extensively drug-resistant bacteria (eXDR), a simple way to assess spontaneous clearance. J. Hosp. Infect..

[10] Kennedy K., Collignon P. (2010). Colonisation with *Escherichia coli* resistant to “critically important” antibiotics: A high risk for international travellers. Eur. J. Clin. Microbiol. Infect. Dis..

[11] Jamrozik E., Selgelid M.J. (2019). Surveillance and control of asymptomatic carriers of drug-resistant bacteria. Bioethics.

[12] Gorwitz R.J., Kruszon-Moran D., McAllister S.K., McQuillan G., McDougal L.K., Fosheim G.E., Jensen B.J., Killgore G., Tenover F.C., Kuehnert M.J. (2008). Changes in the Prevalence of Nasal Colonization with Staphylococcus aureus in the United States, 2001–2004. J. Infect. Dis..

[13] McMillan E.A., Gupta S.K., Williams L.E., Jové T., Hiott L.M., Woodley T.A., Barrett J.B., Jackson C.R., Wasilenko J.L., Simmons M. (2019). Antimicrobial resistance genes, cassettes, and plasmids present in salmonella enterica associated with United States food animals. Front. Microbiol..

[14] Marshall B.M., Levy S.B. (2011). Food animals and antimicrobials: Impacts on human health. Clin. Microbiol. Rev..

[15] Creely K. (2004). Infection Risk and Embalming. Inst Occup Med [Internet]. http://www.ifsa.us/images/Article.IOM.InfectionRisksAndEmbalming.pdf.

[16] World Health Organization (2017). Guidelines for the Prevention and Control of Carbapenem-Resistant Enterobacteriaceae, Acinetobacter baumannii and Pseudomonas aeruginosa in Health Care Facilities.

[17] Magiorakos A.P., Burns K., Rodríguez Baño J., Borg M., Daikos G., Dumpis U., Lucet J.C., Moro M.L., Tacconelli E., Simonsen G.S. (2017). Infection prevention and control measures and tools for the prevention of entry of carbapenem-resistant Enterobacteriaceae into healthcare settings: Guidance from the European Centre for Disease Prevention and Control. Antimicrob. Resist. Infect. Control.

[18] Centers for Diasease Control and Prevention (2015). CDC. Facility Guidance for Control of Carbapenem-Resistant Enterobacteriaceae (CRE). Natl. Cent. Emerg. Zoonotic Infect. Dis..

[19] Clark M., Haisman-Welsh White E., Houliston P., Tagg J. (2018). Infection Prevention and Control and Management of Producing Enterobacteriaceaee: Guidelines for Health Care Providers in New Zealand Acute and Residential Care Facilities. https://www.health.govt.nz/system/files/documents/publications/infection-prevention-control-management-carbapenemase-producing-enterobacteriaceae-dec18.pdf.

[20] Board N.-M.D.H. The Infectious Hazards of Dead Bodies. https://www.nmdhb.govt.nz/dmsdocument/61-the-infectious-hazards-of-dead-bodies.

[21] Demiryürek D., Bayramoǧlu A., Ustaçelebi Ş. (2002). Infective agents in fixed human cadavers: A brief review and suggested guidelines. Anat. Rec..

[22] Bajracharya S., Magar A. (2006). Embalming: An art of preserving human body. Kathmandu Univ. Med. J..

[23] Weed L.A., Baggenstoss A.H. (1951). The Isolation of Pathogens from Tissues of Embalmed Human Bodies. Am. J. Clin. Pathol..

[24] Kabadi C., Smith C., Gomez F. (2013). Potential pathogen transmission on medical student anatomy laboratory clothing. Med. Stud. Res. J..

[25] Sterling T.R., Pope D.S., Bishai W.R., Harrington S., Gershon R.R., Chaisson R.E. (2000). Transmission of Mycobacterium tuberculosis from a Cadaver to an Embalmer. N. Engl. J. Med..

[26] Tabaac B., Goldberg G., Alvarez L., Amin M., Shupe-Ricksecker K., Gomez F. (2013). Bacteria detected on surfaces of formalin fixed anatomy cadavers. Ital. J. Anat. Embryol..

[27] Balta J.Y., Cryan J.F., O’Mahony S.M. (2019). The antimicrobial capacity of embalming solutions: A comparative study. J. Appl. Microbiol..

[28] Balta J.Y., Twomey M., Moloney F., Duggan O., Murphy K.P., O’Connor O.J., Cronin M., Cryan J.F., Maher M.M., O’Mahony S.M. (2019). A comparison of embalming fluids on the structures and properties of tissue in human cadavers. J. Vet. Med. Ser. C Anat. Histol. Embryol..

[29] Thiel W. (1992). The Preservation of the Whole Corpse with Natural Color. Ann. Anat..

[30] Toombs-Ruane L.J., Benschop J., Burgess S., Priest P., Murdoch D.R., French N.P. (2017). Multidrug Resistant Enterobacteriaceae in New Zealand: A Current Perspective. N. Z. Vet. J..

[31] Tumbarello M., Spanu T., Sanguinetti M., Citton R., Montuori E., Leone F., Fadda G., Cauda R. (2006). Bloodstream infections caused by extended-spectrum-β-lactamase- producing Klebsiella pneumoniae: Risk factors, molecular epidemiology, and clinical outcome. Antimicrob. Agents Chemother..

[32] Tumbarello M., Sali M., Trecarichi E.M., Leone F., Rossi M., Fiori B., de Pascale G., D’Inzeo T., Sanguinetti M., Fadda G. (2008). Bloodstream infections caused by extended-spectrum-β-lactamase- producing *Escherichia coli*: Risk factors for inadequate initial antimicrobial therapy. Antimicrob. Agents Chemother..

[33] Moor C.T., Roberts S.A., Simmons G., Briggs S., Morris A.J., Smith J., Heffernan H. (2008). Extended-spectrum β-lactamase (ESBL)-producing enterobacteria: Factors associated with infection in the community setting, Auckland, New Zealand. J. Hosp. Infect..

[34] Williamson D.A., Hefferman H. (2014). The changing landscape of antimicrobial resistance in New Zealand. N. Z. Med. J..

[35] McDougall J. (2020). Aged Residential Care Industry Profile 2019–2020 [Internet]. https://nzaca.org.nz/wp-content/uploads/2020/08/ARC-Industry-Profile-2019-20-Final.pdf.

[36] Starlander G., Melhus Å. (2012). Minor outbreak of extended-spectrum β-lactamase-producing Klebsiella pneumoniae in an intensive care unit due to a contaminated sink. J. Hosp. Infect..

[37] Chia P.Y., Sengupta S., Kukreja A., Sl Ponnampalavanar S., Ng O.T., Marimuthu K. (2020). The role of hospital environment in transmissions of multidrug-resistant gram-negative organisms. Antimicrob. Resist. Infect. Control.

[38] Embil J.M., Dyck B., Plourde P. (2009). Prevention and control of infections in the home. Can. Med. Assoc. J..

[39] Noble W.C., Virani Z., Cree R.G.A. (1992). Co-transfer of vancomycin and other resistance genes from Enterococcus faecalis NCTC 12201 to Staphylococcus aureus. FEMS Microbiol. Lett..

[40] Bosshard P.P., Zbinden R., Abels S., Böddinghaus B., Altwegg M., Böttger E.C. (2006). 16S rRNA gene sequencing versus the API 20 NE system and the VITEK 2 ID-GNB card for identification of nonfermenting Gram-negative bacteria in the clinical laboratory. J. Clin. Microbiol..

[41] Florio W., Baldeschi L., Rizzato C., Tavanti A., Ghelardi E., Lupetti A. (2020). Detection of Antibiotic-Resistance by MALDI-TOF Mass Spectrometry: An Expanding Area. Front. Cell Infect. Microbiol..

[42] Jacob D., Sauer U., Housley R., Washington C., Sannes-Lowery K., Ecker D.J., Sampath R., Grunow R. (2012). Rapid and high-throughput detection of highly pathogenic bacteria by Ibis PLEX-ID technology. PLoS ONE.

[43] Makemson J.C., Fulayfil N.R., Van Ert L. (1998). Differentiation of Marine Luminous Bacteria Using Commercial Identification Plates. Luminescence.

[44] Jones B.V., Marchesi J.R. (2007). Transposon-aided capture (TRACA) of plasmids resident in the human gut mobile metagenome. Nat. Methods.

